# Integrated physiological and multi-omics analyses reveal genotype-specific GABA responses associated with salt tolerance in *Brassica rapa*

**DOI:** 10.3389/fpls.2026.1966891

**Published:** 2026-09-09

**Authors:** Shiyi Li, Yuanyuan Zhang, Zhengnan Xu, Sheng Chen, Yifan Wang, Hao Sun, Lisha Zhang, Junyan Wu, Xiaoyan Liang, Lijun Liu, Wancang Sun, Li Ma

**Affiliations:** 1 State Key Laboratory of Aridland Crop Science/College of Agronomy, Gansu Agricultural University, Lanzhou, China; 2 The UWA Institute of Agriculture, The University of Western Australia, Western Australia, Perth, Australia; 3 State Key Laboratory of Plant Environmental Resilience, College of Biological Sciences, China Agricultural University, Beijing, China

**Keywords:** *Brassica rapa*, GABA, ion flux, proteomics, salt stress, transcriptomics

## Abstract

Soil salinity restricts the yield of winter *Brassica rapa*, yet it remains unclear whether gamma-aminobutyric acid (GABA) alleviates salt stress through universal physiological responses or genotype-specific regulatory patterns. To resolve this question, salt-tolerant KY and salt-sensitive Qin were subjected to four treatments: blank control (CK), sole GABA supplementation(G), single salt stress(S), and combined salt stress plus GABA(S+G). Multiple indicators covering seedling growth status, membrane impairment, reactive oxygen species buildup, antioxidant performance, Na^+^/K^+^ balance and ion transportation were measured. Transcriptome profiling, DIA proteomic detection and qRT-PCR confirmation were also conducted for multi-omics exploration. Exogenous GABA relieved salt-triggered growth suppression, oxidative injury and ion disorder across both genotypes, with KY displaying stronger physiological and molecular responses associated with salt stress adaptation. Under NaCl stress conditions, KY possessed stronger antioxidant buffering potential, steadier Na^+^/K^+^ homeostasis, as well as enhanced native capacities to take in K^+^ and expel Na^+^. Omics data identified a KY-enriched regulatory module associated with phenylpropanoid and flavonoid metabolism, where *BrPAL2*, *BrCSE*, *BrGSTU25*, *BrGASA7* and *BrCYP74A* were identified as candidate genes potentially associated with GABA-responsive salt tolerance. Collectively, GABA exerts non-universal salt-mitigating effects; its protective performance depends on genotype-matched coordination of physiological defense, ionic equilibrium and stress-responsive molecular pathways. These findings provide insights into genotype-dependent GABA responses and may contribute to future evaluation of GABA application and saline-adapted germplasm improvement in *B. rapa*.

## 1 Introduction

As soil salinization continues to expand worldwide, it has become a major environmental constraint that limits agricultural productivity and threatens food security, particularly in arid and semi-arid regions (Munns et al., 2020; FAO, 2024; Irik and Bikmaz, 2024; Wang et al., 2025b). High salinity reduces seed germination, restricts seedling establishment and suppresses biomass accumulation through osmotic stress, ion toxicity and secondary oxidative damage. These effects are especially detrimental during early developmental stages, when young seedlings have limited capacity to maintain water balance, ion selectivity and redox stability. Therefore, understanding the mechanisms underlying plant salt tolerance is essential for improving stress-resilient germplasm and sustaining crop production. *Brassica rapa* L. is an important overwintering oilseed crop in northern and northwestern China, where it contributes to edible oil production and winter-spring soil cover. Its successful production depends on seedling establishment before winter, root-mediated overwintering survival and rapid regreening in spring. However, saline-alkali conditions frequently restrict early growth and biomass accumulation in this crop. Although *B. rapa* shows considerable genotypic variation in stress adaptation, the physiological and molecular basis underlying differences in salt tolerance among winter rapeseed genotypes remains insufficiently understood (Liu et al., 2022; Wu et al., 2022). Improving seedling-stage salt tolerance is therefore directly relevant to stand establishment, overwintering survival, and stable production of winter *B. rapa* in salt-affected regions.

Salt tolerance is not determined by a single protective response, but by the coordination of multiple physiological and molecular processes. Under high-salinity conditions, excessive Na^+^ accumulation disrupts cellular ion balance, competes with K^+^ and Ca^2+^ homeostasis, and interferes with membrane function, photosynthesis and protein metabolism (Zhang et al., 2023; Ramakrishna et al., 2025). Salt-induced osmotic stress further reduces cellular water potential and turgor, while reactive oxygen species (ROS) accumulation causes lipid peroxidation, membrane injury, protein damage and metabolic dysfunction (Yu et al., 2024; Sackey et al., 2025). To cope with these constraints, plants activate a multilayered defence system involving ion exclusion and compartmentalization, osmotic adjustment, antioxidant protection and metabolic remodeling (Tang et al., 2015; Chen et al., 2021; Balasubramaniam et al., 2023; Guo et al., 2025; Ni et al., 2025). Increasing evidence indicates that environmentally friendly external strategies, including bioactive regulators and nanomaterial-assisted approaches, can enhance plant stress resilience by improving antioxidant capacity, regulating physiological processes, and promoting plant growth under adverse environmental conditions (Chen et al., 2025) (Chen et al., 2026) highly interconnected. Ion imbalance can promote ROS production, oxidative damage can impair membrane transport activity, and metabolic remodeling can provide both osmoprotective compounds and antioxidant secondary metabolites. Thus, salt tolerance should be understood as an integrated response linking ion homeostasis, redox regulation and downstream metabolic adaptation.

Gamma-aminobutyric acid (GABA) is a widely distributed four-carbon non-protein amino acid that functions as both a metabolite and a signaling molecule in plant responses to abiotic stress (Yuan et al., 2023; Wang et al., 2024). Previous studies in several plant species have shown that GABA can alleviate salt-induced injury by regulating Na^+^ and K^+^ transport, enhancing antioxidant capacity, maintaining osmotic balance and modulating stress-related metabolism. For example, GABA has been associated with SOS1 and NHX-mediated Na^+^ regulation in tomato, H^+^-ATPase-dependent ion transport in rice, improved ROS scavenging in Arabidopsis and wheat, polyamine-related stress protection in maize, and betaine-associated osmotic adjustment in poplar (Su et al., 2019; Zhang et al., 2019, 2024; Wu et al., 2020; Badr et al., 2024; Wang et al., 2025a, 2025c). These findings suggest that GABA may act at the interface of ion transport, redox buffering and metabolic regulation. However, most previous studies have treated GABA as a broadly effective stress-alleviating compound, whereas whether its protective effect depends on the intrinsic regulatory capacity of different genotypes remains unclear. This issue is particularly relevant for crops such as *B. rapa*, in which natural genotypic variation provides an opportunity to distinguish general stress alleviation from genotype-specific regulatory responses.

Despite increasing evidence for the protective role of GABA, its regulatory framework in *B. rapa* remains poorly resolved. In particular, it is still unclear whether GABA-associated salt stress alleviation involves coordinated regulation across physiological and molecular layers, including ion homeostasis, antioxidant defense and secondary metabolic pathways. Phenylpropanoid and flavonoid metabolism are of particular interest because they contribute to cell wall reinforcement, ROS scavenging and stress-related metabolic adjustment. However, whether these pathways are differentially recruited by GABA in salt-tolerant and salt-sensitive *B. rapa* genotypes has not been systematically examined. Addressing this question requires an integrated approach that connects physiological traits, dynamic ion fluxes and genome-wide molecular responses.

In this study, we used two winter *B. rapa* genotypes with contrasting salt tolerance to investigate the effects of exogenous GABA under salt stress. By integrating physiological measurements, Na^+^ and K^+^ flux analysis, transcriptomics, proteomics and qRT-PCR validation, we aimed to determine whether GABA-associated salt stress alleviation differs between the salt-tolerant genotype KY and the salt-sensitive genotype Qin. We further sought to identify the key physiological processes, molecular pathways and candidate genes associated with the stronger GABA response in the tolerant genotype. This study provides a multi-layered framework for understanding genotype-specific GABA responses under salt stress in winter *B. rapa*. Winter *B. rapa* is a critical oilseed crop cultivated across saline regions of northwest China, and our results can guide field GABA foliar application and stress-resilient rapeseed cultivation under saline soil conditions.

## 2 Materials and methods

### 2.1 Plant materials and growth conditions

Two winter *Brassica rapa* genotypes with contrasting salt tolerance were used in this study. The salt-tolerant genotype SCKY-6-27 (hereafter KY) and the salt-sensitive genotype 197–2018 Qin 10-45 (hereafter Qin) were previously identified from 30 winter rapeseed accessions under salt stress (Ma et al., 2025). Seeds were provided by the Rapeseed Research Team at Gansu Agricultural University. Uniform, healthy seeds were selected and surface-sterilized by immersion in 0.5% sodium hypochlorite for 5 min, followed by five rinses with sterile deionized water. The sterilized seeds were then placed in Petri dishes to allow germination. After 5–7 days of germination, seedlings were transferred to hydroponic chambers containing half-strength Hoagland nutrient solution, which was renewed every 3 days (Ayyaz et al., 2021). At the two-leaf stage, seedlings were randomly assigned to four treatment groups (**Supplementary Table 1**): control treatment (CK), GABA treatment (G), salt treatment (S), and combined salt and GABA treatment (S+G). Salt stress was imposed using 150 mM NaCl, and exogenous GABA was applied at a concentration of 0.5 mM. The concentration of 0.5 mM GABA was selected based on previous reports and preliminary observations showing effective stress alleviating responses without visible growth inhibition (Ahmad and Fariduddin, 2025). GABA solution was sprayed onto the leaves every 2 days until the leaf surface was uniformly wetted. Plants in the CK and S treatments received the same volume of distilled water at the corresponding time points. All seedlings were cultivated in an environmentally controlled chamber under the following conditions: 25°C, 30% relative humidity, a 16 h light/8 h dark cycle, and a light intensity of 6400 lx. Leaf samples were collected on day 12, immediately snap-frozen in liquid nitrogen, and stored at −80°C for subsequent analyses. The hydroponic experiment was performed using a randomized complete block design, and three biological replicates were established for each treatment.

### 2.2 Growth and physiological traits

After 12 days of salt stress and exogenous GABA application, seedlings were selected from each treatment group to measure the following five agronomic traits: seedling height, leaf area, root length, fresh leaf weight, and dry leaf weight. Seedling height (cm) and root length (cm) were measured using a ruler. The leaf area of seedlings was determined using a Li-3100C leaf area meter (LI-COR Inc., Lincoln, USA). Fresh and dry biomass were subsequently measured with an electronic balance accurate to 0.1 mg (Ru et al., 2025). The dry weight of seedlings was determined after drying the samples in an oven at 120°C for 4 hours and then weighing the dried samples. Relative electrical conductivity (REL) of plant leaves under different treatments was measured using a DDS-307A conductivity meter (Shanghai Instrumentation Science & Technology Co., Ltd., Shanghai, China). Healthy leaves with consistent growth status were selected as experimental materials, cut into small segments, and placed in clean test tubes. Subsequently, 20 mL of deionized water was added. The tubes were kept at room temperature for 2 hours to measure the initial conductivity of the solution (C1) and the conductivity of the deionized water (C0). After boiling for 15 minutes, the conductivity (C2) was measured (Si et al., 2020). REL was calculated based on the following formula:


REL (%) = [(C1−C0)(C2−C0)]×100

At 10:00 AM on the 12th day of treatment, leaf chlorophyll content was measured using a portable chlorophyll meter SPAD-502 (Konica Minolta, Inc., Osaka, Japan). To evaluate the effects of salt stress and exogenous GABA application on plasma membrane damage, osmotic regulation, and physiological responses in rapeseed seedlings, the activities of antioxidant enzymes (POD, SOD, and CAT), malondialdehyde (MDA) content, reactive oxygen species (ROS) levels (H_2_O_2_ and O_2_^-^), proline content (Pro), soluble sugar content, soluble protein content, ion concentrations (Na^+^ and K^+^), and the K^+^/Na^+^ ratio were determined. Commercial assay kits were used for these measurements, and all procedures were performed according to the manufacturers’ instructions (Solarbio Life Sciences, Beijing, China) (Ahsan et al., 2021).

Root anatomical characteristics were analyzed using paraffin sectioning. Root samples were collected from five-leaf-stage seedlings of *Brassica rapa* genotypes under normal growth conditions and after 12 days of salt-alkali stress treatment. The roots were washed with double-distilled water, cut into approximately 0.5 cm segments, and fixed in FAA solution (5 mL formalin, 5 mL glacial acetic acid, and 90 mL 70% ethanol) at 4°C. Paraffin sections were prepared through dewaxing, dehydration, safranin-fast green staining, and neutral resin mounting. Sections with a thickness of 8 μm were observed using a Leica DM500 optical microscope. Three biological replicates were analyzed for each treatment.

### 2.3 Analysis of Na^+^ and K^+^ flux rates

Non-destructive micro-sensing technology (MIFE, Microelectrode Ion Flux Measurement) was used to detect Na^+^ and K^+^ flux rates in roots and leaf mesophyll (Liu et al., 2019; Yu et al., 2023). When examining roots, plants were first treated in a 0 or 150 mM NaCl medium for 24 hours. Healthy plants with uniform growth were selected, roots were secured to the bottom of a petri dish, and the corresponding test solution (all groups: Na^+^: 1.0 mM NaCl, 0.1 mM KCl, 0.2 mM MES, pH 5.8; K^+^: 1.0 mM KNO_3_, 0 or 150 mM NaCl, 0.2 mM MES, pH 5.8) was added. After 0.5 h immersion, samples were analyzed. For mesophyll analysis, leaf segments were collected from plants subjected to the corresponding control or NaCl treatment. The lower epidermis of the target leaves was removed to expose the mesophyll tissue, and leaf segments were then equilibrated in the corresponding measuring solution before flux recording. For target leaves, the lower epidermis was removed to expose the mesophyll tissue, and a 0.5 cm × 0.2 cm leaf tissue was sampled. To fix the mesophyll tissue samples, corresponding treatment solution (1.0 mM KNO_3_, 0 or 150 mM NaCl, 0.2 mM MES, pH 5.8) was added to submerge the mesophyll tissue at the bottom of a petri dish. After incubation for 3.5 hours, samples were transferred to the respective test solution (Na^+^: same as root Na^+^ test solution; K^+^: same as treatment solution), followed by 0.5 hours of incubation before detection under the microscope. The Na^+^ or K^+^ flux micro-sensor (Xuyue Co., Ltd., Beijing, China) was positioned at the root elongation zone (950 μm from the root tip apex) or on the surface of the mesophyll tissue. The sensor was maintained 5 μm above the sample surface without contact. Data was recorded for 5 minutes, with 6 biological replicates per group. Flux rate data in pico mol•cm^-2^•s^-1^ were directly read and output via imFluxes V3.0 software (Xuyue Co., Ltd., Beijing, China). The sign of the flux value (positive or negative) indicates the direction of Na^+^ and K^+^ transport: a positive sign indicates Na^+^ and K^+^ flowing from the root cell interior to the root cell exterior (for mesophyll, this means from the mesophyll cell interior to the apoplast), while a negative sign indicates the opposite direction (Li et al., 2022).

### 2.4 Total RNA extraction and RT-qPCR analysis

For RNA extraction, leaves sampled from 12-day-old seedlings were immediately frozen in liquid nitrogen after harvest and stored at −80°C. Total RNA was then extracted with an RNAprep Pure Plant Kit (Tiangen, Beijing, China) according to the manufacturer’s instructions (Yuan et al., 2021), After extraction, RNA concentration and purity were determined using a NanoDrop 2000 spectrophotometer (Thermo Fisher Scientific, Wilmington, NC, USA) (Vanhinsbergh et al., 2022). RNA integrity was assessed using the RNA Nano6000 Assay Kit on an Agilent Bioanalyzer 2100 system (Agilent Technologies, CA, USA) (Fu et al., 2019). Selected transcripts were amplified by RT-qPCR using gene-specific primers synthesized by Sangon Biotech (Shanghai, China). PP2A, which encodes the serine/threonine protein phosphatase PP2A-2 catalytic subunit, was used as the reference gene (**Supplementary Table 2**) (Tao et al., 2023). Experiments were conducted according to the protocol of Tiangen’s FastReal qPCR PreMix (SYBR Green) premix reagent, and relative gene expression levels were calculated using the 2^-ΔΔCT^ method (Zeng et al., 2018). qRT-PCR experiments employed three biological replicates and three technical replicates.

### 2.5 Transcriptome sequencing

For transcriptome sequencing, mRNA libraries were generated using the Hieff NGS Ultima Dual-mode mRNA Library Prep Kit (Yeasen Biotechnology Co., Ltd., Shanghai, China) in accordance with the manufacturer’s guidelines (Fu et al., 2023). After library construction, DNA fragments were purified using AMPure XP magnetic beads (Beckman Coulter, Beverly, USA). Library integrity and quality were subsequently examined with an Agilent 2100 Bioanalyzer system. Sequencing was performed using an Illumina HiSeq X Ten Sequencer (OeBiotech Co., Ltd., Shanghai, China) for 24 samples, including two genotypes, four treatments per genotype and three biological replicates per treatment, generating 150 bp paired-end reads. The *Brassica rapa* ‘Longyou 7’ genome was used as the reference genome (Wu et al., 2022). Raw reads were filtered to remove adaptor sequences, low-quality reads and reads containing ambiguous bases. Clean reads were then mapped to the *Brassica rapa* ‘Longyou 7’ reference genome using HISAT2. Gene expression levels were quantified using featureCounts and normalized as FPKM values. Differentially expressed genes were identified using DESeq2, with an adjusted *p* value < 0.05 and |log_2_ fold change| ≥ 1 as the significance threshold. GO and KEGG enrichment analyses were performed using clusterProfiler, and terms with an adjusted *p* value < 0.05 were considered significantly enriched. Gene set enrichment analysis was conducted based on ranked gene expression changes to evaluate pathway-level responses.

### 2.6 Proteomics analysis

Leaf samples were collected and rapidly frozen in liquid nitrogen, then stored at −80°C until protein extraction. Proteomic analysis was performed by OeBiotech (Shanghai, China) using a data independent acquisition based quantitative proteomic strategy. After protein extraction, proteins were digested with trypsin, and the resulting peptides were analysed by liquid chromatography tandem mass spectrometry using a Thermo Scientific Orbitrap Astral mass spectrometer. In the DIA acquisition mode, precursor ions within defined mass to charge windows and their fragment ions were systematically scanned, allowing high coverage and reproducible quantification of peptides across samples. All mass spectrometry data were merged and processed using DIA-NN software for database searching and DIA based protein quantification. The protein sequence database used for searching was Brapa.protein.fasta. Protein abundance was estimated based on peptide peak areas or signal intensities. The PSM and protein false discovery rates were both controlled at 1%. Differentially abundant proteins (DAPs) were selected for further analysis based on statistical significance (*p* < 0.05) and fold change (> 1.2 or < 0.83) (Orsburn, 2021; Hu et al., 2025). Detected proteins were functionally annotated using the GO (Ashburner et al., 2000) and KEGG (Ashburner et al., 2000; Kanehisa and Goto, 2000) databases.

### 2.7 Statistical analysis

Phenotypic and physiological data were analyzed using SPSS 25.0 (IBM, Inc., Armonk, NY, USA). Physiological traits were analyzed using two-way or three-way analysis of variance (ANOVA), with genotype, salt treatment, and GABA application as fixed factors. When significant main effects or interaction effects were detected, multiple comparisons were performed using Tukey’s HSD test at *p* < 0.05. Data visualization was performed using GraphPad Prism 10.0 (GraphPad Software, USA, www.graphpad.com) and OriginPro 2021 (OriginLab, USA). Statistical significance was determined at a threshold of *p* ≤ 0.05. Physiological data are shown as the mean ± SD from no fewer than three independent biological replicates, whereas ion flux data are shown as the mean ± SE from six biological replicates unless otherwise indicated.

## 3 Results

### 3.1 Effects of salt stress and exogenous GABA on seedling growth and root structure of winter rapeseed

**Figure 1:**
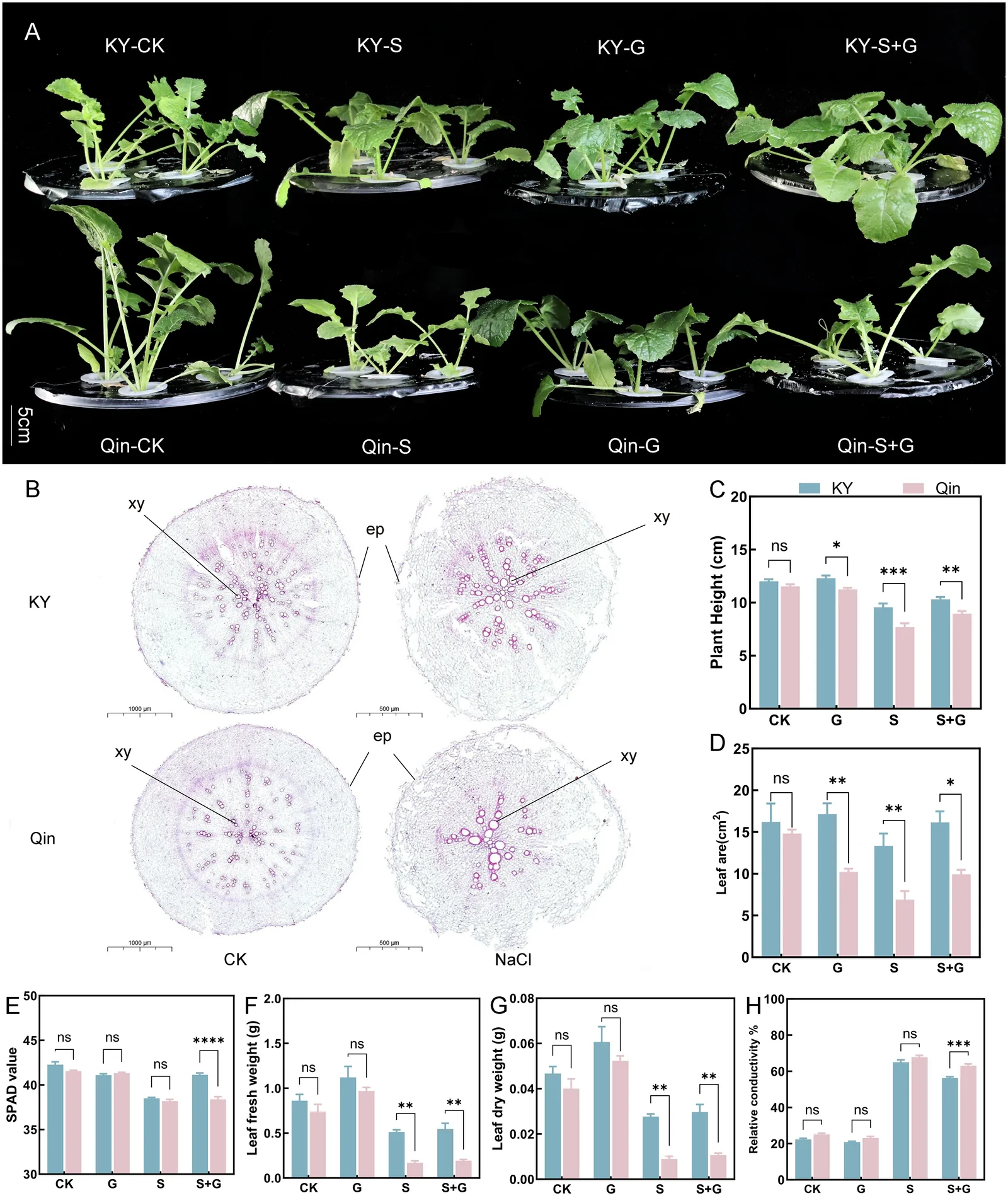
Effects of salt stress and exogenous GABA on seedling growth and root anatomical structure in two winter *B. rapa* genotypes. KY (SCKY-6-27) is the salt-tolerant genotype, and Qin (197–2018 Qin 10-45) is the salt-sensitive genotype. **(A)** Representative phenotypes of KY and Qin under control (CK), GABA alone (G), salt stress (S), and salt stress plus GABA (S+G) treatments. **(B)** Representative root cross-sections of KY and Qin under control and NaCl treatments, showing salt-induced anatomical alterations. xy, xylem vessel; ep, epidermis. **(C)** Plant height. **(D)** Leaf area. **(E)** SPAD value. **(F)** Fresh leaf weight. **(G)** Dry leaf weight. **(H)** Relative electrical conductivity. Data in panels **(C–H)** are presented as mean ± SD of three independent biological replicates. Statistical significance between treatments is indicated as follows: ns, no significant difference; *p < 0.05; **p < 0.01; ***p < 0.001; ****p < 0.0001.

Salt stress markedly inhibited seedling growth in both winter *Brassica rapa* genotypes, with stronger visible growth suppression in the salt-sensitive genotype Qin than in the salt-tolerant genotype KY. Phenotypic observations showed that NaCl treatment caused wilting, leaf curling, and growth retardation in both genotypes, whereas Qin displayed more pronounced leaf necrosis and overall growth inhibition. Exogenous GABA application visibly alleviated these symptoms and improved seedling vigour under saline conditions (**Figure 1A**; **Supplementary Figure S1**). At the anatomical level, salt stress also disrupted root structure (**Figure 1B**). Under control conditions, both genotypes showed relatively intact root cross-sections, with continuous epidermal and cortical tissues and clearly organized vascular cylinders. After NaCl treatment, the root sections became structurally disordered, with irregular outlines, partial epidermal damage, loosened cortical tissues, and disturbed cellular organization around the stele. These anatomical alterations were more evident in Qin, whereas KY retained comparatively clearer tissue organization.

Quantitative measurements further supported the phenotypic observations (**Figures 1C–H**). Under salt stress, plant height decreased by 21.3% in KY and 34.2% in Qin, while leaf area declined by 17.7% and 53.5%, respectively, indicating stronger growth inhibition in the sensitive genotype. SPAD values also decreased under NaCl treatment in both genotypes. Fresh weight was reduced by 40.4% in KY and 30.8% in Qin, whereas dry weight declined by 40.8% and 77.5%, respectively. Although the reduction in fresh weight was not greater in Qin than in KY, Qin showed stronger decreases in plant height, leaf area and dry weight, indicating more severe impairment of seedling development and dry matter accumulation. Relative electrical conductivity increased markedly after salt treatment and remained higher in Qin than in KY. Exogenous GABA partially improved plant height, leaf area, SPAD value, and seedling biomass under salt stress, while also reducing relative electrical conductivity in both genotypes. Overall, these results indicate that salt stress severely restricted seedling growth in both genotypes, with stronger inhibitory effects in Qin, whereas exogenous GABA partially alleviated these changes and showed a more evident protective effect in KY.

### 3.2 Effects of GABA on physiological indicators under NaCl stress

**Figure 2:**
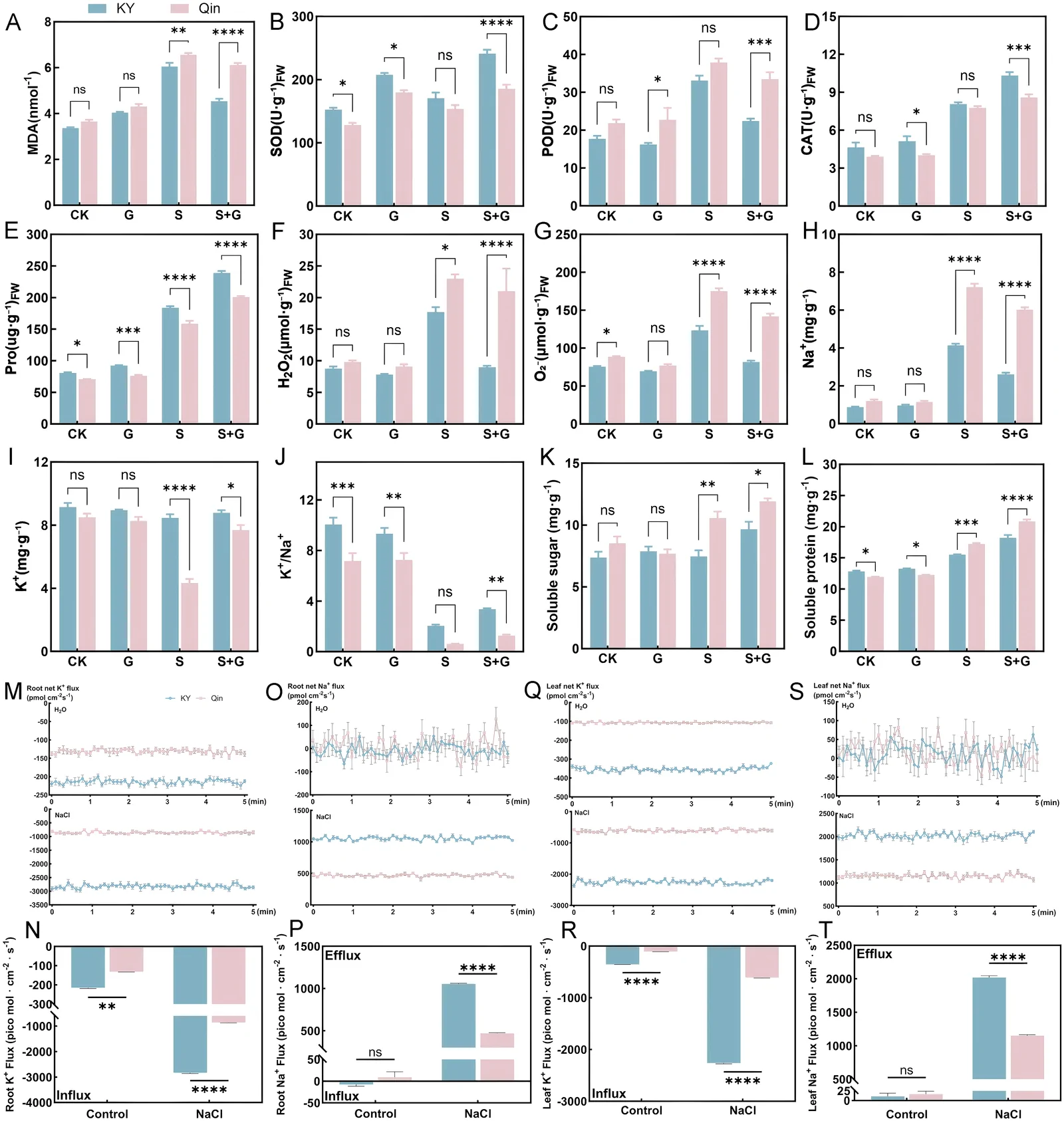
Physiological responses to exogenous GABA and ion flux characteristics under salt stress in two winter *B. rapa* genotypes. **(A)** MDA content. **(B)** SOD activity. **(C)** POD activity. **(D)** CAT activity. **(E)** Pro content. **(F)** H_2_O_2_ content. **(G)** O_2_^-^ content. **(H)** Na^+^ content. **(I)** K^+^ content. **(J)** K^+^/Na^+^ ratio. **(K)** Soluble sugar content. **(L)** Soluble protein content. **(M, N)** Net K^+^ flux rate and average K^+^ flux in the root elongation zone under control and NaCl stress conditions. **(O, P)** Net Na^+^ flux kinetics and mean Na^+^ flux in the root elongation zone under control and NaCl treatment conditions. **(Q, R)** Net K^+^ flux kinetics and mean K^+^ flux across the leaf mesophyll surface under control and NaCl stress. **(S, T)** Net Na^+^ flux kinetics and mean Na^+^ flux across the leaf mesophyll surface under control and NaCl stress. Panels **(A–L)** show physiological responses under CK, G, S and S+G treatments, whereas panels **(M–T)** show ion fluxes under control and NaCl conditions. Values in panels **(A–L)** are shown as the mean ± SD (n = 3), whereas data in panels **(M–T)** are expressed as the mean ± SE (n = 6). Positive flux values indicate efflux, and negative values indicate influx. Statistical differences among treatments are denoted using the following annotations: ns, not significant, *p* > 0.05; *p < 0.05; **p < 0.01; ***p < 0.001; and ****p < 0.0001.

To assess the physiological effects of exogenous GABA under salt stress, we measured indicators related to membrane damage, antioxidant activity, ROS accumulation, osmotic adjustment, and ion homeostasis in KY and Qin (**Figure 2**). NaCl stress induced severe oxidative damage. Compared with their respective CKs, Qin exhibited a more pronounced increase in MDA content under NaCl stress: Qin-S MDA content increased by 82.1% compared to Qin-CK, while KY-S MDA content increased by 61.9% compared to KY-CK. Following GABA application to alleviate salt stress, MDA content in KY decreased by 35.3% compared to the NaCl-treated control. In contrast, MDA content in Qin decreased by only 22.5% relative to the NaCl-treated control and remained 38.7% higher than the CK. These results indicate that GABA more effectively alleviated salt-induced membrane injury in KY than in Qin.

Antioxidant enzyme activities were also strongly affected by salt stress and GABA treatment. Under NaCl stress, SOD, POD, and CAT activities increased in both genotypes, but the magnitude of induction was consistently greater in KY. SOD activity increased by 99.1% in KY-S and 65.3% in Qin-S relative to their respective controls, and GABA further increased SOD activity by 25.6% in KY and 15.8% in Qin compared with the corresponding salt-stressed treatment. Similarly, POD activity increased by 200.0% in KY-S and 130.0% in Qin-S, and was further enhanced by GABA by 33.3% and 20.0%, respectively. CAT activity showed the same trend, increasing by 192.9% in KY-S and 120.0% in Qin-S, with an additional increase of 52.4% in KY-S+G and 30.0% in Qin-S+G. Overall, antioxidant enzyme activities increased in both genotypes under salt stress, and the GABA-induced enhancement was more pronounced in KY.

Salt stress also caused marked ROS accumulation. H_2_O_2_ and O_2_- levels increased significantly in both genotypes, with Qin showing higher H_2_O_2_ accumulation than KY. Exogenous GABA reduced ROS levels under saline conditions in both genotypes, and the reduction in H_2_O_2_ was more evident in KY. In addition, osmotic adjustment-related indicators, including proline and other compatible solutes, were altered by salt stress and GABA treatment (**Figures 2E, K, L**). These results suggest that GABA contributed to physiological protection not only by limiting oxidative damage but also by promoting osmotic adjustment under salt stress.

Salt stress severely disrupted ion homeostasis, leading to increased Na^+^ accumulation, reduced K^+^ status, and a sharp decline in the K^+^/Na^+^ ratio. Qin showed higher Na^+^ accumulation and a more pronounced decrease in the K^+^/Na^+^ ratio than KY under NaCl treatment. Exogenous GABA partially alleviated ionic imbalance by reducing Na^+^ accumulation and restoring the K^+^ to Na^+^ ratio in both genotypes, although the extent of recovery differed between KY and Qin. However, even after GABA treatment, the K^+^/Na^+^ ratio remained significantly lower than that of the controls, indicating incomplete recovery of ionic balance. Overall, salt stress increased membrane damage, ROS accumulation, and ionic disturbance in both genotypes, whereas exogenous GABA partially alleviated these physiological changes. The protective effects of GABA were more pronounced in KY, particularly in antioxidant regulation and maintenance of ion homeostasis.

### 3.3 Na^+^ and K^+^ fluxes in root and mesophyll tissues under salt stress

To further evaluate genotype dependent ion transport responses under saline conditions, we measured net Na^+^ and K^+^ fluxes in the root elongation zone and leaf mesophyll using non destructive microelectrode ion flux measurement (MIFE) (**Figures 2M–T**). Because positive values indicate ion movement from the cell interior to the external medium, whereas negative values indicate movement in the opposite direction, the direction of flux was interpreted as efflux or influx according to the sign of the recorded value.

For K^+^ flux, both root and mesophyll tissues exhibited net K^+^ influx under control and NaCl conditions. In roots, KY showed stronger net K^+^ influx than Qin under control conditions. NaCl treatment markedly enhanced root K^+^ influx in both genotypes, with the average influx reaching approximately 2700 to 3000 pmol cm^-2^ s^-1^ in KY and 800 to 1000 pmol cm^-2^ s^-1^ in Qin (**Figures 2M, N**). A similar pattern was observed in leaf mesophyll tissues. Under NaCl stress, KY maintained a substantially stronger net K^+^ influx than Qin, reaching approximately 2200 to 2400 pmol cm^-2^ s^-1^ in KY and 500 to 700 pmol cm^-2^ s^-1^ in Qin (**Figures 2Q, R**). These results indicate that KY had a stronger inward K^+^ flux response than Qin under saline conditions.

For Na^+^ flux, both genotypes showed only minor fluctuations around zero under control conditions, and no significant difference was detected between KY and Qin. In contrast, NaCl treatment induced clear net Na^+^ efflux in both root and mesophyll tissues. In roots, the average Na^+^ efflux reached approximately 1000 to 1100 pmol cm^-2^ s^-1^ in KY, compared with approximately 400 to 500 pmol cm^-2^ s^-1^ in Qin (**Figures 2O, P**). In leaf mesophyll tissues, Na^+^ efflux was also significantly stronger in KY, reaching approximately 2000 pmol cm^-2^ s^-1^, whereas Qin showed approximately 1100 to 1200 pmol cm^-2^ s^-1^ under the same NaCl treatment (**Figures 2S, T**).

Overall, the two genotypes showed distinct ion flux responses under salt stress. KY exhibited stronger net K^+^ influx and Na^+^ efflux than Qin in both root and mesophyll tissues. These flux characteristics are consistent with the physiological results showing lower Na^+^ accumulation, higher K^+^ status and a more favorable K^+^ to Na^+^ balance in KY under salt stress. Thus, the superior salt tolerance of KY may be partly associated with its stronger capacity for dynamic K^+^ acquisition and Na^+^ exclusion under saline conditions.

### 3.4 Transcriptome profiling reveals genotype-dependent reprogramming under salt stress and GABA treatment

#### 3.4.1 Global transcriptome structure distinguishes KY from Qin under salt stress and GABA treatment

To characterize genotype-dependent transcriptional responses to salt stress and GABA application, RNA-seq was performed on 24 leaf samples representing two genotypes and four treatments. A total of 166.9 Gb of clean data was generated, with Q30 values ranging from 97.18% to 97.68%, and more than 89.13% of reads mapped to the reference genome, indicating high sequencing quality and reliable transcriptome coverage (**Supplementary Table 3**).

**Figure 3:**
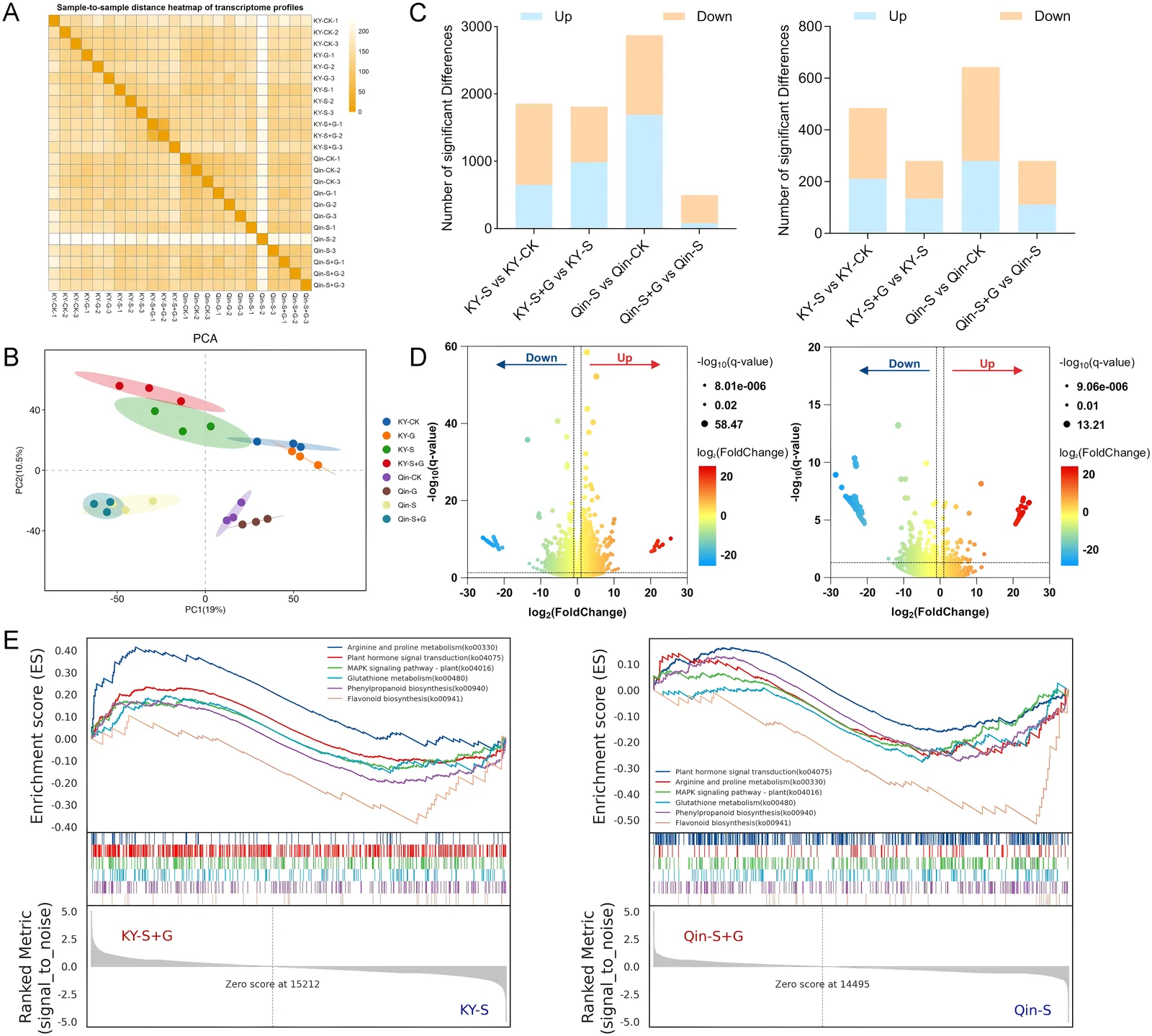
Transcriptome profiling reveals a more defence oriented GABA responsive pattern in KY than in Qin under salt stress. **(A)** Sample-to-sample distance heatmap of transcriptome profiles across all 24 samples. **(B)** Principal component analysis (PCA) of transcriptome samples. **(C)** Numbers of significantly up and down regulated genes in the major pairwise comparisons, including KY-S vs KY-CK, KY-S+G vs KY-S, Qin-S vs Qin-CK, and Qin-S+G vs Qin-S. **(D)** Volcano plots showing differentially expressed genes in KY-S+G vs KY-S (left) and Qin-S+G vs Qin-S (right). Each point represents one gene; the x-axis indicates log_2_(fold change), and the y-axis indicates -log_10_(q-value). **(E)** Gene set enrichment analysis (GSEA) of representative stress- and defence-associated pathways in KY-S+G vs KY-S (left) and Qin-S+G vs Qin-S (right), including arginine and proline metabolism, plant hormone signal transduction, MAPK signaling pathway, glutathione metabolism, phenylpropanoid biosynthesis, and flavonoid biosynthesis.

Sample-to-sample distance analysis showed strong clustering of biological replicates within each treatment, supporting the reproducibility of the dataset (**Figure 3A**). Principal component analysis further separated samples according to both genotype and treatment, indicating that salt stress and GABA application induced substantial but structured transcriptomic variation in both genotypes (**Figure 3B**). These results support subsequent comparative transcriptome analysis between KY and Qin.

#### 3.4.2 GABA induces distinct transcriptional response patterns in KY and Qin

Differential expression analysis showed that the two genotypes differed not only in DEG number but also in the distribution of GABA-responsive pathways. Under salt stress, 651 genes were upregulated and 1785 genes were downregulated in KY, whereas 1693 genes were upregulated and 1177 genes were downregulated in Qin. Following GABA treatment under salt stress, 1334 genes were upregulated and 666 genes were downregulated in KY-S+G versus KY-S, whereas 1785 genes were upregulated and 856 genes were downregulated in Qin-S+G versus Qin-S (**Figures 3C, D**). These results indicate that Qin showed a larger number of DEGs, whereas KY exhibited a comparatively smaller but distinct transcriptional response. To clarify the overlapping characteristics of differentially expressed genes between KY and Qin under salt stress, a Venn diagram analysis was performed to examine the distribution of DEGs in both varieties after stress treatment (**Supplementary Figure 2A**; **Supplementary Table 4**).

GO enrichment analysis revealed that, in the comparison between the KY-S+G and KY-S groups, upregulated differentially expressed genes were mainly enriched in processes related to phenylpropanoid metabolism, suberin biosynthesis, and hormone responses, and were also associated with functions involving the cell wall and water transport (**Supplementary Table 5**). In contrast, in the comparison between the Qin-S+G and Qin-S groups, upregulated differentially expressed genes were primarily enriched in processes related to putrescine and terpenoid metabolism, protein transport, and photosynthesis, indicating distinct functional response patterns between the two genotypes under salt stress (**Supplementary Table 6**). Thus, compared with KY, Qin showed less prominent enrichment of GABA-responsive DEGs in defence-associated pathways (**Supplementary Figures 2B, C**). KEGG enrichment analysis revealed that, in the comparison between the KY-S+G and KY-S groups, differentially expressed genes were significantly enriched in pathways related to phenylpropanoid biosynthesis and arginine and proline metabolism, suggesting that cell wall reinforcement and osmotic adjustment play important roles in the stress response. In contrast, in the comparison between the Qin-S+G and Qin-S groups, differentially expressed genes were significantly enriched in pathways associated with photosynthesis, particularly photosynthesis-antenna proteins, as well as carbon and nitrogen metabolism, alpha-linolenic acid metabolism, and phenylpropanoid biosynthesis (**Supplementary Figures 2D, E**). These results suggest that, after GABA application under salt stress, Qin showed stronger enrichment in photosynthesis and primary metabolic processes, whereas KY displayed more prominent enrichment of defence associated pathways.

Pathway enrichment analysis further revealed genotype-dependent differences in the biological distribution of GABA-responsive genes. In KY, these genes were mainly enriched in plant hormone signal transduction, MAPK signaling, glutathione metabolism, phenylpropanoid biosynthesis, and flavonoid biosynthesis (**Figure 3E**). In contrast, Qin showed weaker enrichment in these defence-associated pathways and a broader distribution across general metabolic categories. Together, these results indicate that GABA induced distinct pathway-level transcriptional responses in KY and Qin.

### 3.5 Transcriptome-based pathway reconstruction reveals stronger shikimate, phenylpropanoid, and flavonoid pathway responses in KY

#### 3.5.1 Carbon precursor supply and shikimate pathway responses are more continuous in KY

**Figure 4:**
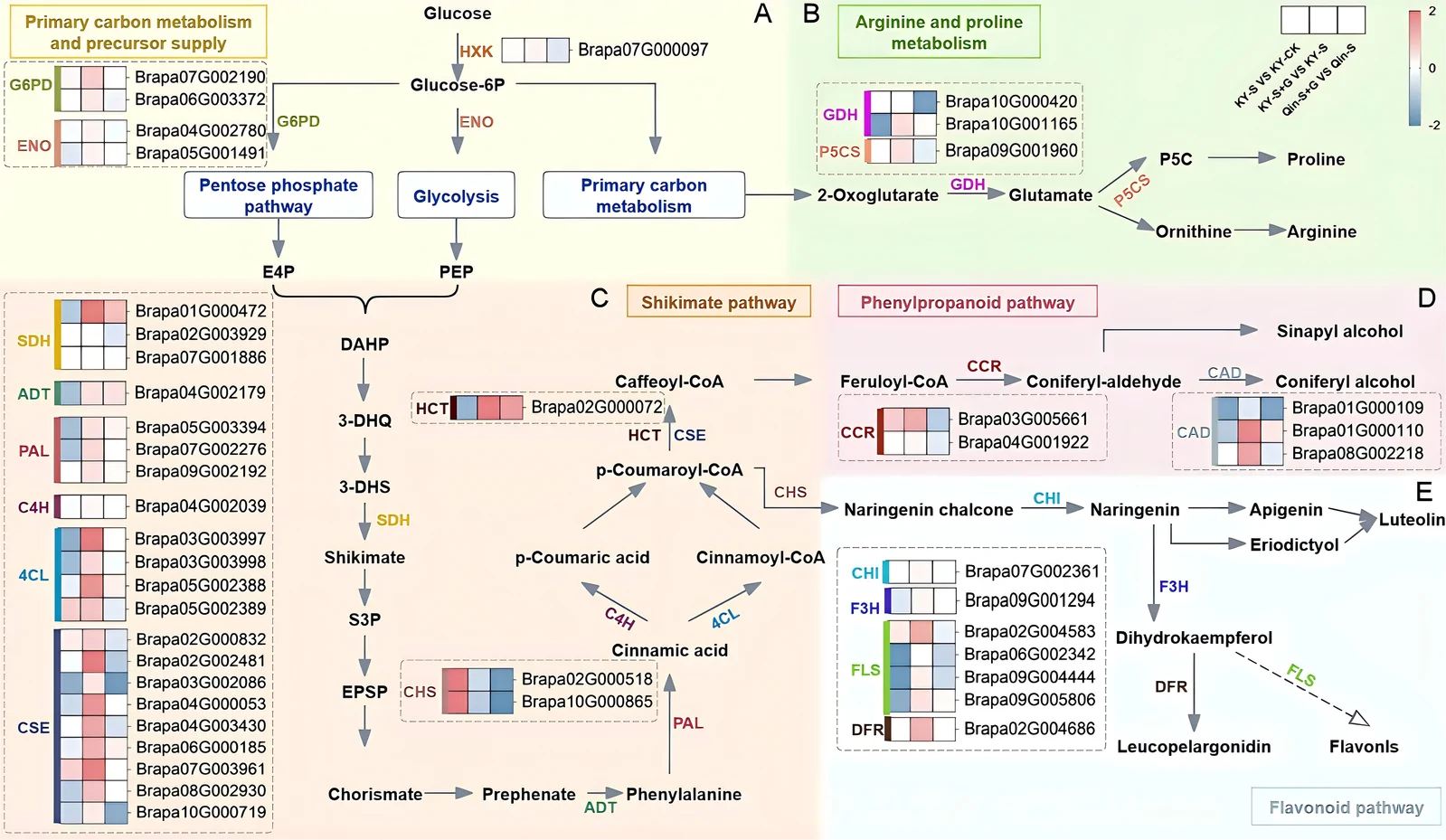
Differences in transcript abundance between KY and Qin in metabolic pathways related to primary carbon metabolism, shikimate metabolism, phenylpropanoid biosynthesis, flavonoid biosynthesis, and arginine/proline metabolism under salt stress and GABA treatment. **(A)** Primary carbon metabolism and precursor supply pathway. **(B)** Arginine and proline metabolism-related pathway. **(C)** Shikimate pathway. **(D)** Phenylpropanoid metabolism-related pathway. **(E)** Flavonoid metabolism-related pathway. In each mini-heatmap, each row represents a gene, while the three columns represent the comparisons KY-S vs KY-CK, KY-S+G vs KY-S, and Qin-S+G vs Qin-S. Red and blue indicate relative transcript changes based on log_2_ fold change, representing up- and down-regulation, respectively. Enzyme annotations: HXK, Hexokinase; G6PD, Glucose-6-phosphate dehydrogenase; ENO, Enolase; SDH, Shikimate dehydrogenase; ADT, Arogenate dehydratase; PAL, Phenylalanine ammonia-lyase; C4H, Cinnamate 4-hydroxylase; 4CL, 4-Coumarate: CoA ligase; HCT, Hydroxycinnamoyl-CoA:shikimate hydroxycinnamoyl transferase; CSE, Caffeoyl shikimate esterase; CCR, Cinnamoyl-CoA reductase; CAD, Cinnamyl alcohol dehydrogenase; CHS, Chalcone synthase; CHI, Chalcone isomerase; F3H, Flavanone 3-hydroxylase; FLS, Flavonol synthase; DFR, Dihydroflavonol 4-reductase; GDH, Glutamate dehydrogenase; P5CS, Δ1-Pyrroline-5-carboxylate synthetase.

To clarify how the transcriptomic response was channelled into downstream metabolism, we reconstructed the pathway from primary carbon metabolism to shikimate, phenylpropanoid and flavonoid biosynthesis (**Figures 4A–E**). This analysis showed that GABA responsive transcriptional changes in KY were not randomly distributed across metabolism, but were organized along a transcriptional axis connecting primary carbon metabolism with secondary metabolic pathways. Genes associated with the pentose phosphate pathway and glycolysis were potentially related to the supply of E4P and PEP, two precursors that feed into the shikimate pathway. In turn, the shikimate branch linked upstream carbon metabolism to phenylalanine formation, thereby providing a metabolic entry point for stress-responsive secondary metabolism.

In KY, several key steps along this upstream route showed coordinated transcript accumulation, suggesting that GABA was associated not only with downstream defence related transcriptional responses, but also with the activation of genes potentially involved in precursor supply. By contrast, the corresponding response in Qin was weaker and less continuous across the pathway. Thus, the genotype difference was expressed not simply at the level of terminal defence genes, but also at the level of transcriptional activation of genes linking primary carbon metabolism with aromatic amino acid biosynthesis.

#### 3.5.2 GABA is associated with stronger transcriptional responses of phenylpropanoid and flavonoid-related genes in KY

Downstream of phenylalanine, the reconstructed pathway highlighted the phenylpropanoid backbone and its flavonoid branch as the most distinctive GABA-responsive modules in KY (**Figure 4E**).Core phenylpropanoid steps, including PAL, C4H, 4CL and CSE associated nodes, showed coordinated transcriptional induction in KY, suggesting enhanced expression of genes involved in phenylalanine metabolism and downstream phenylpropanoid-related processes. This pattern was particularly evident for *BrPAL2* and *BrCSE*, which emerged as representative transcriptional nodes in the tolerant genotype.

The flavonoid branch downstream of p-coumaroyl-CoA showed a similar genotype-dependent pattern. CHS, CHI and F3H associated steps were more strongly activated in KY, indicating that GABA was associated not only with activation of the phenylpropanoid backbone, but also with stronger transcriptional responses in flavonoid related branches. In contrast, the corresponding transcriptional changes in Qin were comparatively weak, discontinuous, or confined to only part of the pathway. These results suggest that a major divergence between the two genotypes lies in the extent to which GABA is associated with transcriptional regulation of phenylpropanoid and flavonoid-related pathways. In KY, this pathway showed a more continuous transcriptional response, whereas in Qin the same metabolic branch was only partially responsive.

### 3.6 Modular and co-expression analysis support a KY biased defence associated transcriptional response under GABA treatment

#### 3.6.1 Expression trend clustering reveals distinct GABA responsive expression patterns in KY and Qin

**Figure 5:**
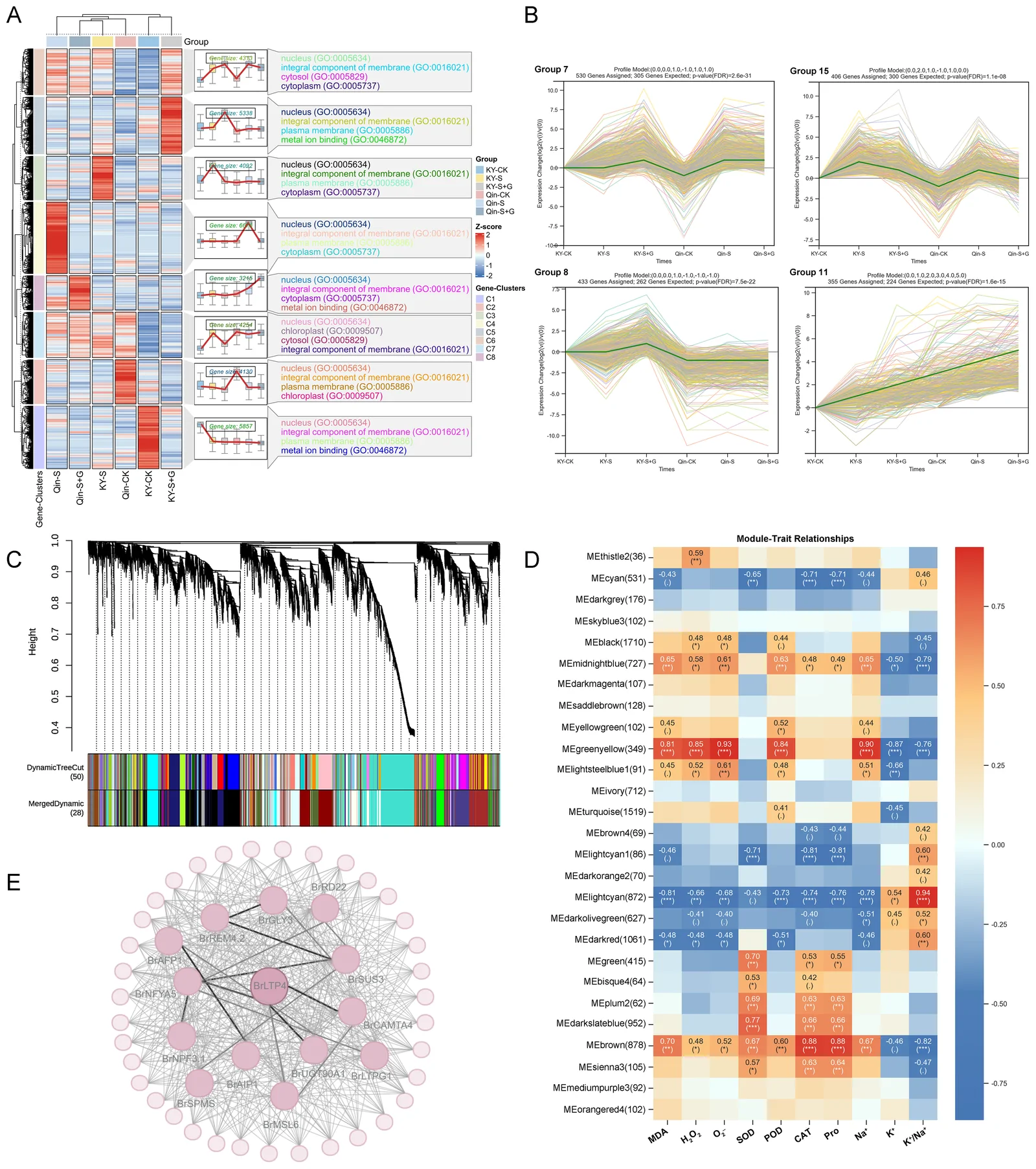
Trend clustering and co-expression analyses support a KY biased defence associated transcriptional response under GABA treatment. **(A)** Global clustering heatmap of DEGs across all treatments, showing major expression trends and associated functional enrichments. **(B)** K-means clustering of differentially expressed genes reveals distinct expression responses between the two genotypes under different treatments. **(C)** Hierarchical clustering dendrogram and dynamic tree cutting used for module identification in weighted gene co-expression network analysis (WGCNA). **(D)** Module–trait relationship heatmap showing correlations between co-expression modules and physiological traits, including MDA, H_2_O_2_, O_2_-, SOD, POD, CAT, proline, Na^+^, K^+^, and K^+^/Na^+^. Values in each cell indicate correlation coefficients and corresponding significance levels. **(E)** Representative hub-gene interaction network from a key co-expression module associated with the coordinated stress response. *p < 0.05; **p < 0.01; ***p < 0.001.

To further resolve how transcriptomic responses were organized within each genotype, we examined the expression trajectories of all DEGs across the treatment series. K-means clustering grouped the responsive genes into eight major clusters (C1–C8), revealing clear genotype-dependent differences in response organisation (**Figure 5A**; **Supplementary Table 7**). Although Qin showed a larger overall DEG burden under salt stress and GABA treatment, the functional orientation of these changes differed markedly from that of KY. In KY, clusters C6 and C7, comprising 3,080 genes, showed an active induction pattern, characterised by moderate activation under salt stress followed by a further increase after GABA treatment. The identified clusters were mainly enriched in stress-responsive pathways, including plant hormone signal transduction, MAPK signaling, and phenylpropanoid biosynthesis. This pattern suggests that GABA enhanced the coordination of defence-associated transcriptional regulation in KY.

By contrast, the corresponding response in Qin was less coherent. Clusters C2 and C5, comprising 1,415 genes, displayed a passive recovery pattern, with strong downregulation under salt stress followed by only partial rebound after GABA application. These clusters were enriched mainly in basal metabolic categories such as butyrate metabolism and β-alanine metabolism, rather than in the major defence-associated pathways observed in KY. Thus, the key difference between the two genotypes was not simply the scale of transcriptional change, but the degree to which GABA responsive genes were associated with defence related functions. In KY, the transcriptional response was channelled towards signaling and secondary metabolism, whereas in Qin it remained comparatively dispersed and metabolically non-specific.

To refine this pattern further, DEGs were additionally partitioned into 16 expression groups (**Figure 5B**; **Supplementary Figure S3**). Among them, Groups 7 and 15 showed similar trajectories and were enriched in phenylpropanoid biosynthesis, jasmonate biosynthesis and sulfotransferase activity, and included representative genes such as *Brapa02G002481* (*BrCSE*), *Brapa02G003137* (*BrCYP74A*) and *Brapa03G004312* (*BrGASA7*). In contrast, Groups 8 and 11 were associated mainly with amino acid, fatty acid, starch and sucrose metabolism. These results suggest that the GABA-responsive program in KY was preferentially assembled around defence-associated metabolic functions, whereas the Qin response was more closely associated with broad metabolic adjustment.

#### 3.6.2 Co-expression analysis links transcriptional coordination to the stress-alleviation phenotype

Weighted gene co-expression network analysis (WGCNA) was performed to assess the network-level organization of genotype-dependent transcriptional changes. Based on FPKM-normalized expression values and a soft-thresholding power of β = 12, the resulting network satisfied the scale-free topology criterion, and 26 co-expression modules were identified by hierarchical clustering. (**Figures 5C, D**). The resulting module trait relationships suggested that GABA responsive expression changes were associated with coordinated gene modules rather than only isolated gene level changes.

Importantly, this network structure was consistent with the trend-clustering results described above. In KY, the GABA-responsive program was associated with modules enriched in defence-related functions and showed close correspondence with the physiological alleviation phenotype, including improved ion homeostasis and stronger antioxidant performance. By contrast, Qin lacked comparable activation of such defence-associated transcriptional modules and instead retained a more diffuse expression structure. Thus, both trend based clustering and co expression analysis support an association between the KY biased GABA response and defence related transcriptional modules, whereas the Qin response appeared broader and less functionally focused. The co-expression network revealed several highly connected genes within the key module, including genes associated with stress responses, transcriptional regulation, carbohydrate metabolism and membrane-related processes. Among them, *BrLTP4* showed relatively high connectivity, suggesting that membrane-associated genes may participate in the GABA-mediated response to salt stress (**Figure 5E**).

### 3.7 Proteomic profiling and cross omics analysis support a KY biased candidate defence associated module

#### 3.7.1 Proteomic profiling supports GABA responsive defence related pathway remodeling

Proteomic analysis was performed to determine whether the transcriptional defence program was accompanied by changes at the protein level. The identified peptides were mainly 7–20 amino acids in length, indicating reliable proteomic quality for downstream analysis (**Supplementary Figure 4A**). Under salt stress, 211 and 280 proteins were upregulated, whereas 274 and 363 proteins were downregulated in KY and Qin, respectively. After GABA application under salt stress, 312 and 233 proteins were upregulated, while 403 and 382 proteins were downregulated in KY and Qin, respectively (**Supplementary Figure 4B**).

Functional enrichment analysis showed that salt- and GABA-responsive DAPs were mainly associated with oxidative stress responses, heme binding, glutathione-related activity, and metabolic processes. KEGG enrichment further highlighted phenylpropanoid biosynthesis, flavonoid biosynthesis, α-linolenic acid metabolism, arginine and proline metabolism, sulfur metabolism, glutathione metabolism, and cutin, suberin and wax biosynthesis. Notably, phenylpropanoid biosynthesis and flavonoid-related metabolism were repeatedly enriched, supporting the transcriptome-based observation that GABA-responsive molecular remodeling converged on secondary metabolism and stress-defence pathways (**Supplementary Figures 4C–F**; **Supplementary Tables 8, 9**). These proteomic results therefore provided an additional molecular layer supporting the involvement of phenylpropanoid/flavonoid-associated defence processes in GABA-mediated salt-stress alleviation.

**Figure 6:**
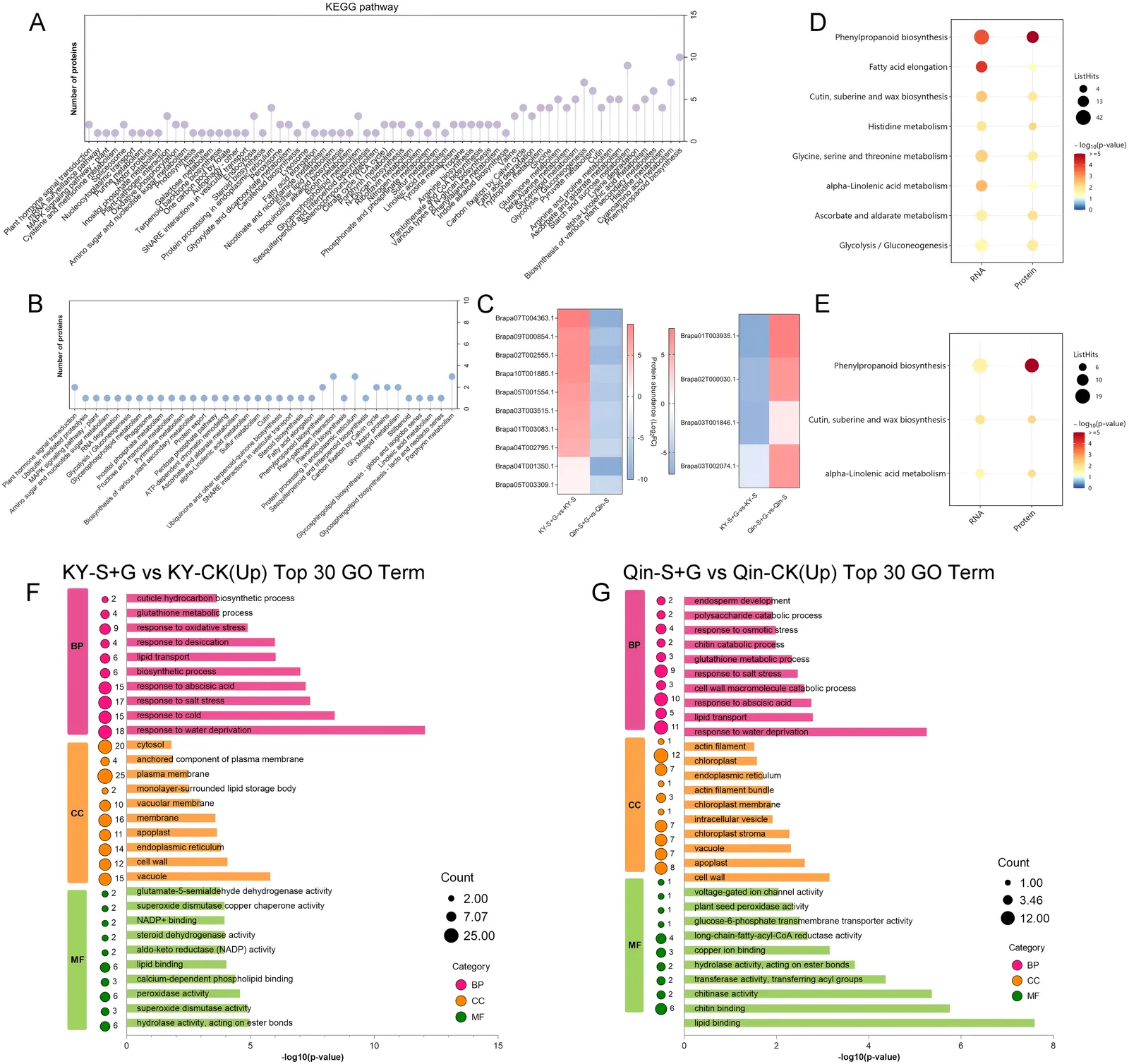
Proteome GSEA and integrated transcriptome proteome analysis of GABA associated salt stress responses in KY and Qin. **(A)** Number of proteins enriched in different KEGG pathways in the salt-tolerant genotype KY under salt stress (S) and combined salt and GABA treatment (S+G), as determined by proteome-based proteins set enrichment analysis. **(B)** Number of proteins enriched in different KEGG pathways in the salt-sensitive genotype Qin under S and S+G treatments. **(C)** Heatmaps showing the abundance changes of proteins involved in GABA-mediated salt stress alleviation in KY and Qin. The color scale represents log_2_FC values. **(D, E)** Integrated transcriptome and proteome KEGG enrichment analysis in KY **(D)** and Qin **(E)**. Bubble color indicates the −log_10_(p-value), and bubble size represents the number of enriched genes or proteins, referred to as ListHits. **(F, G)** Top 30 enriched GO terms of up-regulated genes in KY **(F)** and Qin **(G)** under S+G treatment compared with the control condition. GO terms are classified into biological process (BP), cellular component (CC), and molecular function (MF).

Comparative protein abundance analysis further suggested a genotype-dependent pattern. KY showed higher abundance of several stress-related proteins, including glutathione S-transferase, leucine-rich repeat-containing protein, mRNA cap-binding protein and phenylalanine ammonia-lyase, whereas Qin showed relatively higher abundance of proteins related to basal metabolic regulation. These results suggest that the protein-level response in KY was more closely linked to antioxidant regulation, stress signalling and secondary metabolic defence, while Qin displayed a broader but less defence-oriented proteomic adjustment (**Figures 6A–C**).

#### 3.7.2 Cross-omics concordance is stronger and more focused in KY than in Qin

**Figure 7:**
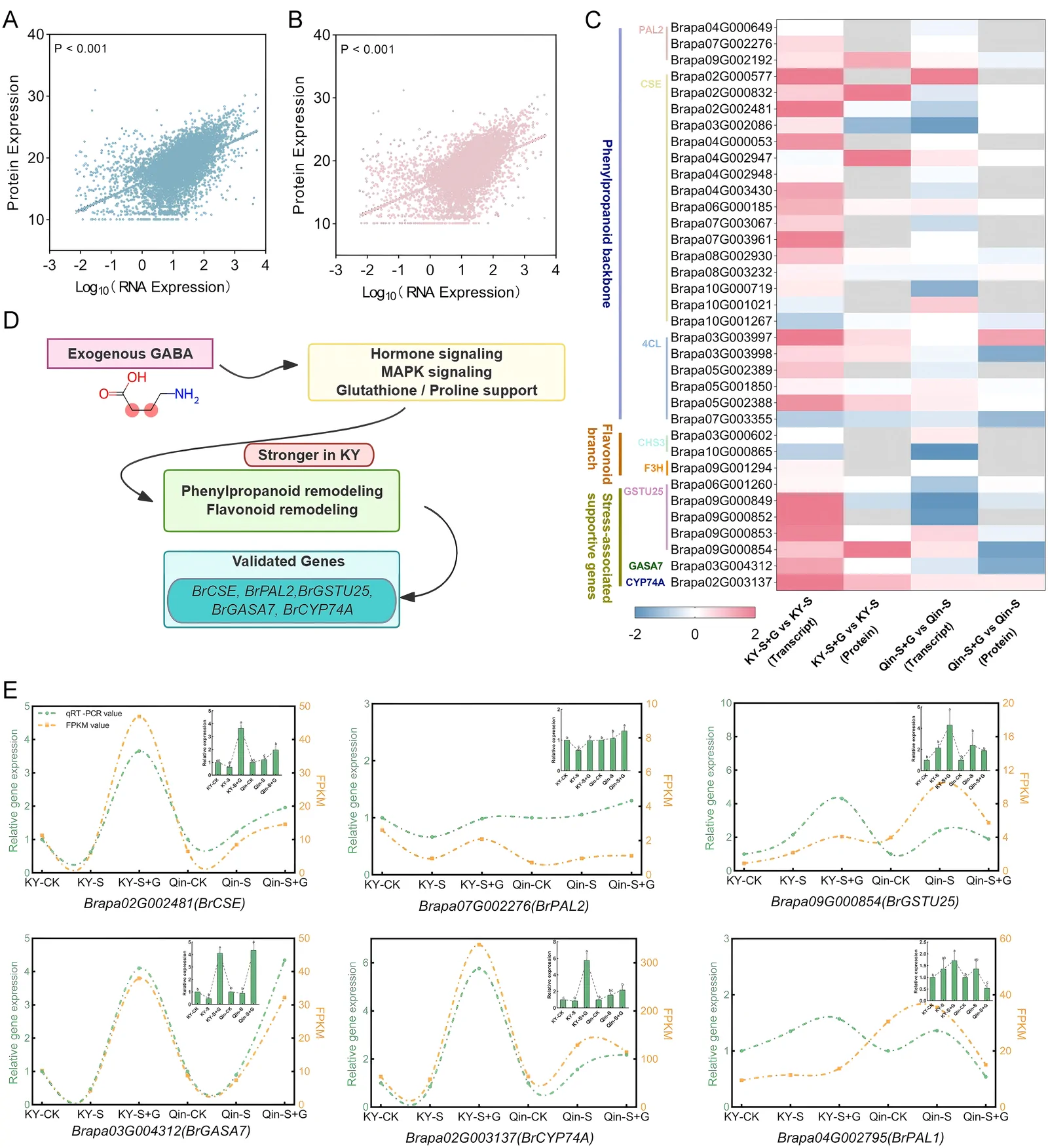
Cross omics and qRT-PCR validation support a KY biased candidate defence associated module under salt stress and GABA treatment. **(A)** Global correlation between transcript abundance and protein abundance in KY. **(B)** Global correlation between transcript abundance and protein abundance in Qin under the corresponding comparison. **(C)** Heatmap of representative genes involved in the phenylpropanoid backbone, flavonoid branch, and stress-associated supportive processes, displaying transcript and protein responses in KY-S+G vs KY-S and Qin-S+G vs Qin-S. **(D)** Working model summarizing genotype specific responses associated with GABA mediated salt stress alleviation, highlighting the stronger signaling, antioxidant support, and phenylpropanoid/flavonoid remodeling observed in KY. **(E)** qRT-PCR validation of representative genes. The dashed lines represent qRT-PCR relative expression trends, and the inset bar plots show the corresponding relative transcript abundance.

To determine whether the transcriptional patterns identified above were accompanied by corresponding changes at the protein level, we integrated the transcriptome and proteome datasets for the key salt and GABA-responsive contrasts. In both genotypes, transcript and protein changes showed overall positive relationships, suggesting that transcript level changes were partly associated with proteome remodeling under salt stress and GABA treatment. However, the cross-omics response was more coherent in KY than in Qin. In KY, a larger fraction of responsive molecules showed concordant changes across transcript and protein levels, whereas Qin displayed a weaker and less focused pattern of coordination, consistent with a less efficiently organized stress response (**Figures 7A, B**).

Pathway-centered integration further showed that molecules displaying concordant responses in KY were preferentially associated with phenylpropanoid biosynthesis and related defence functions. By contrast, the corresponding Qin response contained a higher proportion of molecules with weaker or less coordinated behavior across omics layers. These data indicate that the difference between the two genotypes extends beyond transcript abundance alone and includes a higher capacity in KY to translate transcriptional reprogramming into a more consistent downstream molecular response. Thus, the tolerant genotype not only activates stress-defence pathways more strongly, but also maintains greater transcript-to-protein continuity within those pathways. (**Figures 6D–G**, **7C**; **Supplementary Tables 10, 11**).

#### 3.7.3 Representative genes support a KY biased phenylpropanoid and flavonoid related response

We next examined a set of representative genes selected from the pathway and network analysis to validate the KY-biased response at higher resolution (**Figure 7D**). qRT-PCR analysis showed that genes associated with phenylpropanoid remodeling, antioxidant support and stress-related signaling generally followed the RNA-seq trends and displayed clearer induction in KY than in Qin. In particular, *Brapa02G002481* (*BrCSE*) and *Brapa07G002276* (*BrPAL2*), two representative genes in the phenylpropanoid pathway, were strongly induced in KY-S+G, whereas their responses in Qin were weaker and more variable. Similarly, the antioxidant-related gene *Brapa09G000854* (*BrGSTU25*) and the signaling-associated genes *Brapa03G004312* (*BrGASA7*) and *Brapa02G003137* (*BrCYP74A)* showed more stable and coordinated responses in the tolerant genotype (**Figure 7E**).

Correlation analysis between qRT-PCR and RNA-seq further supported this genotype difference. The agreement between the two platforms was stronger in KY, indicating that its transcriptional response was not only more focused at the pathway level but also more reproducible at the level of representative genes. By contrast, Qin showed weaker concordance between qRT-PCR and RNA-seq, consistent with the broader but less coordinated response pattern described above. These validation results support a KY biased candidate defence associated module involving phenylpropanoid and flavonoid related responses, together with antioxidant and signaling related genes. This axis appears to be reinforced by GABA in the tolerant genotype but only weakly assembled in the sensitive genotype (**Supplementary Figure 5**).

Taken together, the proteomic, cross omics and qRT-PCR results support a candidate defence associated module in KY involving phenylpropanoid and flavonoid related responses, together with glutathione metabolism, hormone signaling and stress related regulatory genes. This module appears to be specifically reinforced by GABA in the salt-tolerant genotype, whereas the corresponding response in Qin is weaker, more dispersed and less consistently transmitted across molecular layers.

## 4 Discussion

The combined physiological and multi-omics analysis presented here indicate that the key difference between KY and Qin lies not simply in stress injury severity, but in whether GABA can effectively channel the response towards a coordinated defence program.

### 4.1 GABA induces a genotype-dependent defence program rather than a uniform salt-relief response

Salt stress simultaneously imposes osmotic, ionic, and oxidative constraints on plant growth, and successful acclimation depends on how efficiently these layers are integrated into a coherent defence program rather than on the magnitude of any single response alone (Munns et al., 2020; Balasubramaniam et al., 2023; Yu et al., 2024). In this context, GABA should not be viewed simply as a generic stress mitigator. Previous work in Arabidopsis and tomato has shown that GABA can improve salinity tolerance by promoting cytosolic K^+^ retention, reducing Na^+^ accumulation, and strengthening antioxidant metabolism (Wu et al., 2020; Yu et al., 2024). Our results extend this concept to *B. rapa* and further show that the critical difference between KY and Qin lies not merely in injury severity, but in whether GABA can effectively reorganize the stress response into a focused and coordinated protective program. This research supplies a theoretical basis for differentiated GABA foliar application on diverse rapeseed cultivars in saline agricultural fields.

### 4.2 Antioxidant buffering and membrane protection are early components of the GABA response

The physiological data indicate that antioxidant buffering and membrane stabilization form an early and necessary layer of the GABA response. Salt stress increased membrane injury and ROS accumulation in both genotypes, whereas GABA partially restored membrane stability and enhanced SOD, POD, and CAT activities, with a stronger effect in KY. This interpretation is consistent with previous studies showing that exogenous or enhanced endogenous GABA alleviates salinity damage by reducing ROS accumulation and activating antioxidant systems in *Arabidopsis* and tomato (Wu et al., 2020; Wang et al., 2024; Yu et al., 2024). These observations support the view that GABA contributes to stress tolerance in part through redox buffering and membrane protection.

However, the present results also suggest that antioxidant recovery alone does not fully explain genotype-dependent tolerance. If redox buffering were the sole determinant, the gap between KY and Qin should have narrowed substantially after GABA treatment. Instead, Qin retained higher membrane injury and weaker recovery, indicating that antioxidant support in the sensitive genotype was only partially translated into downstream protective output. Thus, in KY, GABA appears to act not only as a promoter of antioxidant defence, but also as a coordinator that links redox protection to broader stress-adaptive reprogramming.

### 4.3 Ion homeostasis is a major physiological output of the coordinated KY response

Maintenance of K^+^/Na^+^ homeostasis emerged as another major point of divergence between the two genotypes. KY maintained higher tissue K^+^ levels and a more favorable K^+^ to Na^+^ balance, while MIFE analysis further showed stronger net K^+^ influx and Na^+^ efflux under NaCl stress in both roots and leaves, whereas Qin accumulated more Na^+^ and exhibited a sharper decline in K^+^/Na^+^ balance. The central role of Na^+^ exclusion and compartmentation in salinity adaptation is well established, with SOS1 functioning as a major plasma membrane Na^+^/H^+^ antiporter and vacuolar sequestration contributing to intracellular Na^+^ detoxification (Zhang et al., 2023; Ramakrishna et al., 2025). Moreover, GABA has been shown to act upstream of H^+^-ATPase, thereby facilitating membrane potential maintenance, K^+^ retention and reduced Na^+^ uptake under salinity (Su et al., 2019). Our data are consistent with this framework and suggest that the superior ionic balance of KY is an important physiological component associated with its stronger response to GABA under salt stress. The superior ionic regulation trait of KY can be exploited to breed new salt-resistant oilseed rapeseed varieties adapted to saline farmland in Northwest China.

Importantly, ion homeostasis in KY should not be interpreted as an isolated transport phenotype. Rather, it appears to be embedded within a broader response architecture in which signaling competence, membrane protection, and downstream metabolic adjustment are more tightly coupled Qin, by contrast, responded to GABA at the level of physiology, but failed to translate that response into a comparably stable ionic defence state. This distinction may explain why GABA improved ionic status in both genotypes, yet only in KY did that improvement become part of a more coherent salt-tolerance program.

### 4.4 Phenylpropanoid and flavonoid related pathways represent a candidate downstream module in the KY response

One important outcome of the present study is that GABA treatment in KY was associated with stronger transcriptional and proteomic responses in pathways linking primary carbon metabolism with phenylpropanoid and flavonoid related processes. This observation extends the interpretation of GABA associated salt alleviation beyond general physiological protection and points to a candidate downstream pathway that may contribute to genotype specific tolerance. Phenylpropanoid metabolism is widely recognized as a major interface between development and plant-environment interaction, contributing to cell wall reinforcement, antioxidant defence, and stress adaptation (Dong and Lin, 2021). More recent studies further show that salt tolerance can be enhanced through flavonoid-related pathways, including transcriptional regulation of flavonoid biosynthesis and functional enhancement of F3′H-dependent metabolism (Ni et al., 2025; Wu et al., 2026). Our pathway reconstruction and cross omics results are consistent with this conceptual framework.

In KY, the coordinated induction of *BrPAL2*, *BrCSE*, CHS/F3H associated steps, and other representative defence-related genes indicates that GABA was associated with coordinated transcriptional and proteomic changes along the phenylalanine–phenylpropanoid–flavonoid response axis. Such an arrangement may contribute to tolerance through complementary effects: reinforcement of structural barriers and tissue stability on the one hand, and potentially increased capacity for antioxidant related secondary metabolism on the other. In this regard, current models of salinity adaptation increasingly recognize the importance of cell wall remodeling and stress-responsive secondary metabolism as integral components of salt acclimation, rather than passive consequences of injury (Bawa et al., 2026). Qin was not completely unresponsive to GABA, but its limitation was the inability to assemble these changes into a continuous and defence-oriented metabolic program. Thus, the difference between the two genotypes may lie not only in whether GABA triggers a response, but also in the extent to which this response is associated with phenylpropanoid and flavonoid related pathways. Exogenous GABA-mediated modulation of phenylpropanoid-related responses may represent a potential strategy for improving salt tolerance of winter rapeseed under saline conditions.

Despite the above findings, this study has several limitations. All transcriptomic and proteomic analysis were based on leaf samples, and root responses to GABA under salt stress need further investigation. Moreover, key metabolites of phenylpropanoid and flavonoid pathways were not detected, and the functions of *BrPAL2* and *BrCSE* require in-depth validation. For practical production, the effects of GABA should be assessed under field saline-alkali conditions. Future research will focus on genotype-specific GABA management, salt-tolerant germplasm screening and molecular marker development for the genetic improvement of winter *B. rapa*.

## 5 Conclusion

**Figure 8:**
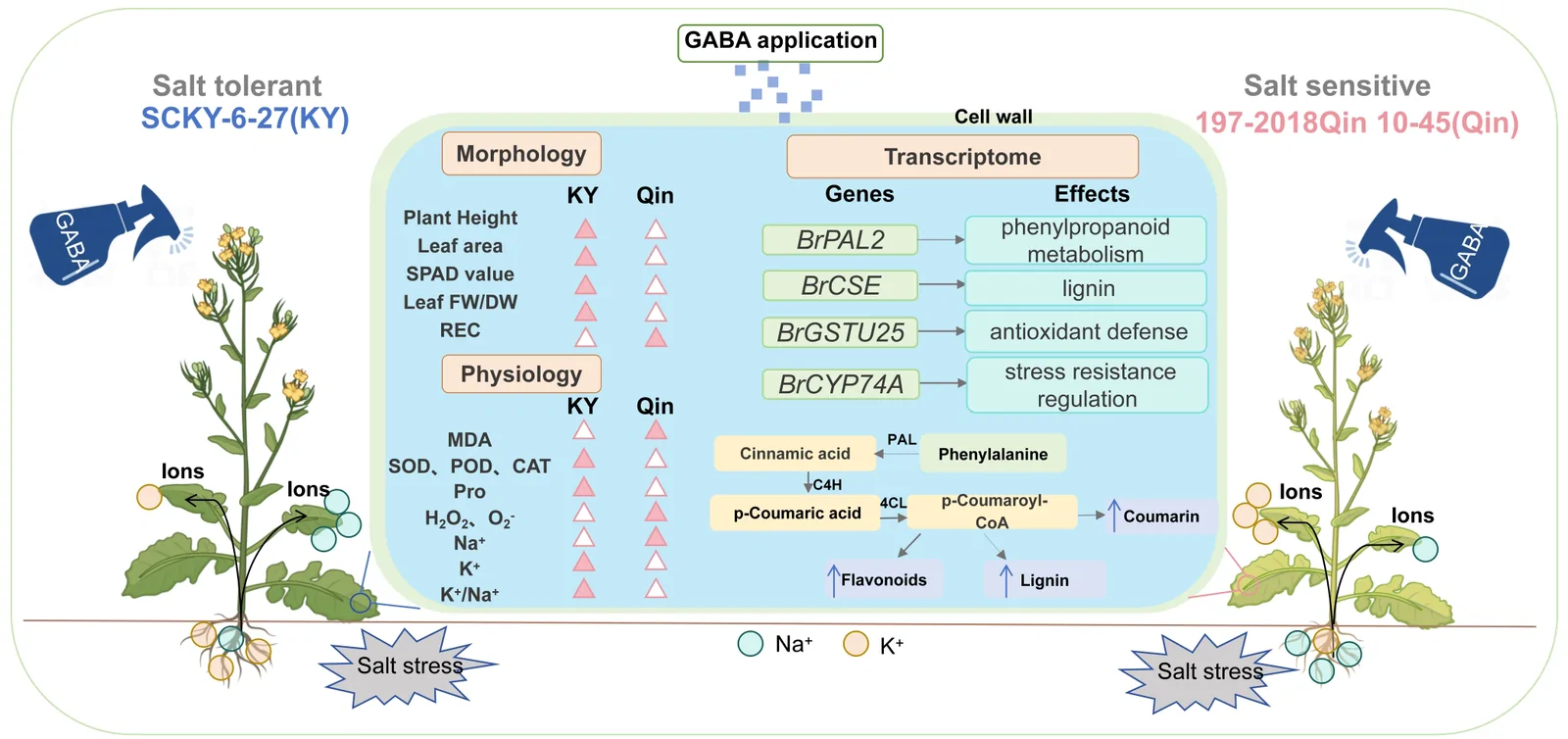
Working model of genotype specific physiological and molecular responses to exogenous GABA under salt stress in winter *B. rapa*. Color intensity indicates the relative magnitude of the response. Blue arrows indicate responses that were more pronounced in KY.

This study demonstrates that exogenous GABA alleviates salt-induced growth inhibition, oxidative damage and ionic imbalance in two winter *B. rapa* genotypes, although the extent and organization of this response differed markedly between them. In the salt tolerant genotype KY, GABA treatment was associated with a more coordinated protective pattern, characterized by stronger antioxidant buffering, improved potassium and sodium homeostasis, enhanced sodium efflux and more focused activation of phenylpropanoid and flavonoid related pathways. The representative genes *BrPAL2*, *BrCSE*, *BrGSTU25*, *BrGASA7* and *BrCYP74A* may therefore provide useful candidate nodes for future functional validation (**Figure 8**).

These findings suggest that GABA does not act as a uniform stress relieving factor across contrasting *B. rapa* genotypes. Rather, its protective effect under salt stress appears to rely, at least in part, on the ability of the tolerant genotype to integrate physiological protection with defence related transcriptional and proteomic responses. The phenylpropanoid and flavonoid associated module proposed here provides a plausible framework for this genotype biased response, but its causal role will require further verification through targeted metabolite profiling and functional analysis of key genes. Collectively, our findings lay a solid foundation for rational GABA field application and genetic improvement of salt tolerance in winter oilseed rapeseed grown in saline northwest China.

## Data Availability

The RNA sequencing data presented in this study have been deposited in the NCBI Sequence Read Archive (SRA) under BioProject accession number PRJNA1522288.
