# Supplementary material for: Integrated physiological and multi-omics analyses reveal genotype-specific GABA responses associated with salt tolerance in Brassica rapa
Source: Front Plant Sci. 2026 Sep 9;17:1966891. doi: 10.3389/fpls.2026.1966891 (PMC13597390; doi:10.3389/fpls.2026.1966891)
Supplement: Supplementary file 1 [file SupplementaryFile2.docx]

## Supporting Information

**Article title:** Regulatory network analysis of GABA mediated salt tolerance in *Brassica rapa* and identification of the key gene *BrPAL2*

**Authors: Shiyi Li^a^, Yuanyuan Zhang^a^, Zhengnan Xu^a^, Sheng Chen^b^, Yifan Wang^a^, Hao Sun^a^, Lisha Zhang^a^, Junyan Wu^a^_,_ Xiaoyan Liang^c^, Lijun Liu^a^, Wancang Sun^a^, Li Ma^a*^**

**Supplementary Figure 1.** Effects of salt stress and exogenous GABA on the growth of KY(SCKY-6-27, salt-tolerant variety) and Qin (197-2018 Qin 10-45, salt-sensitive variety).

**Supplementary Figure 2.** Transcriptome Analysis. (A) Differential gene Veen diagram. (B-C) GO enrichment term plots. (D-E) KEGG enrichment analysis significance bubble plots.

**Supplementary Figure 3.** K-means clustering of all differentially expressed genes (DEGs) across two winter Brassica rapa genotypes (KY and Qin) under treatments. Each line shows the expression pattern of a single DEG, and the green line indicates the mean expression trend of the cluster. The x-axis represents different treatments, and the y-axis indicates log₂(fold change) in expression level.

**Supplementary Figure 4** Proteomics Analysis. (A) Peptide length distribution chart, (B) Differential protein statistics chart. (C-D) Comparison of up and down-regulated entries in InterPro enrichment analysis. (E-F) KEGG enrichment analysis significance bubble chart.

**Supplementary Figure 5.** Validation of candidate genes by quantitative real-time PCR (qRT-PCR). (A-D) The outer plots show the curve-type trend graphs of qRT-PCR values (green) and FPKM values (orange), with the bar charts representing their corresponding expression levels, the inner plots display the bar charts of qRT-PCR values only. (E-F) Correlation analysis of expression levels between qRT-PCR and RNA-Seq for the selected DEGs in KY and Qin. Significant differences were determined by one-way analysis of variance (ANOVA) followed by Tukey’s multiple comparison test, and different lowercase letters indicate significant differences among treatments.
